# Assessment of medial coronoid disease in 180 canine lame elbow joints: a sensitivity and specificity comparison of radiographic, computed tomographic and arthroscopic findings

**DOI:** 10.1186/s12917-015-0556-9

**Published:** 2015-09-25

**Authors:** A. Villamonte-Chevalier, H. van Bree, BJG Broeckx, W. Dingemanse, M. Soler, B. Van Ryssen, I. Gielen

**Affiliations:** Department of Animal Medicine and Surgery, University of Murcia, Murcia, Spain; Department of Veterinary Medical Imaging and Small Animal Orthopaedics, Ghent University, Ghent, Belgium; Laboratory for Pharmaceutical Biotechnology, Ghent University, Ghent, Belgium

**Keywords:** Arthroscopy, Computed tomography, Elbow joint, Medial coronoid disease, Radiography

## Abstract

**Background:**

Diagnostic imaging is essential to assess the lame patient; lesions of the elbow joint have traditionally been evaluated radiographically, however computed tomography (CT) has been suggested as a useful technique to diagnose various elbow pathologies. The primary objective of this study was to determine the sensitivity and specificity of CT to assess medial coronoid disease (MCD), using arthroscopy as gold standard. The secondary objective was to ascertain the radiographic sensitivity and specificity for MCD compared with CT.

**Methods:**

For this study 180 elbow joints were assessed, of which 141 had been examined with radiography, CT and arthroscopy; and 39 joints, had radiographic and CT assessment. Sensitivity and specificity were calculated for CT and radiographic findings using available statistical software.

**Results:**

Sensitivity and specificity of CT using arthroscopy as gold standard resulted in high values for sensitivity (100 %) and specificity (93 %) for the assessment of MCD. For the radiographic evaluation, a sensitivity of 98 % and specificity of 64 - 69 % using CT as the technique of reference, were found.

**Discussion:**

These results suggest that in case of doubt during radiographic assessment, CT could be used as a non-invasive technique to assess the presence of MCD.

**Conclusion:**

Based on the high sensitivity and specificity obtained in this study it has been considered that CT, rather than arthroscopy, is the preferred noninvasive technique to assess MCD lesions of the canine elbow joint.

## Background

Medial coronoid disease (MCD), the most frequently diagnosed component of the elbow dysplasia pathology group, comprises fragmentation or fissuring of the medial coronoid process, and pathological cartilage and/or subchondral bone [1, 2]. MCD is the most common cause of thoracic limb lameness in large- and giant-breed dogs [3].

Diagnostic imaging is essential to assess the lame patient [4]. Elbow joint lesions have traditionally been evaluated radiographically [5–7] with an estimated sensitivity range from 10 to 62 % [1, 8, 9]. Typical MCD findings with radiography include an indistinct and/or deformed contour of the medial coronoid process; an irregular/reduced bone opacity of the medial coronoid process; sclerosis of the distal section of the semilunar notch; and loss of trabecular pattern [5, 10, 11]. Arthrosis, which is often the only radiographic finding associated with MCD [12], is scored as mild, moderate or severe, according to the size of evident osteophytes [13]. For screening purposes, the International Elbow Working Group (IEWG) established as mandatory a medio-lateral flexed projection of each elbow joint and highly recommended an additional cranio-caudal view [13]. However, other researchers recommended three projections as ideal: flexed and extended medio-lateral views and a cranio-caudal oblique view [10].

Computed tomography (CT) [5, 14, 15], magnetic resonance imaging (MRI) [5, 16], nuclear scintigraphy [17] and micro-single photon emission tomography [18] have all been suggested for the diagnosis of various elbow pathologies. However, these techniques are expensive and often only available at referral centres. Computed tomography was found to be more sensitive than radiography for detecting elbow dysplasia, as CT signs are significantly associated with arthroscopic features of elbow dysplasia lesions in dogs [19]; moreover it proved to be the best imaging technique for detecting and measuring elbow incongruity [20, 21] because of the possibility to observe features related with severe elbow incongruity and concomitant FCP (fragmented coronoid process), which were not found in normal joints or congruent joints affected by FCP [20].

In human medicine, arthroscopy is considered the gold standard for evaluating joint cartilage lesions [22]. In veterinary medicine, it is considered the gold standard for assessing MCD [8, 19], because articular surfaces can be evaluated directly and cartilage lesions can be detected. This study’s primary objective was to determine the sensitivity and specificity of CT for assessing MCD using arthroscopy as the gold standard. The secondary objective was to compare the radiographic and CT sensitivity and specificity for detecting MCD. It was hypothesized that CT findings would have a strong correlation to the arthroscopic results.

## Methods

### Study design

Data between January 2010 and December 2011 were collected retrospectively from the patient database of the Department of Veterinary Medical Imaging and Small Animal Orthopaedics of the Ghent University. Permission to access the database was given by the head of the department Prof. Dr. Henri van Bree. Inclusion criteria were bilateral radiographic, CT and, if available, arthroscopic assessment of the elbow joints.

### Radiographic technique

Using a digital radiography system, EDR6 (digital radiographic system) EKLIN device from Canon (Canon Medical Systems), three standard radiographic views—a lateral extension, lateral flexion and a 15° oblique cranio-medial caudo-lateral—were taken of each elbow joint in the dogs [1, 10].

### CT technique

Dogs were anaesthetized using propofol (Rapinovet, Schering-Plough) in a bolus of 2 mg/kg of body weight administered intravenously and then intubated. Anaesthesia was maintained with isoflurane (IsoFlo, Abbott Laboratories) and 100 % oxygen. CT images of both elbow joints were obtained with a 4-slice scanner (LightSpeed, GE Medical systems) using 120 kVp, 140 mA and 25 cm FOV parameters. Dogs were placed in left lateral recumbent position with both thoracic limbs extended symmetrically cranially and the head pulled out of the gantry to avoid artefacts [14]. Contiguous transverse images 1.3 mm thick were obtained from the proximal aspect of the olecranon to 2 cm distal to the elbow joint using a bone algorithm. DICOM files of each scan were retrieved and analysed using work-station software (eFilm, Merge, Merge eMed).

### Arthroscopic technique

An experienced surgeon (BVR) performed arthroscopic assessment using a 2.4 mm, 25° oblique arthroscope (Richard Wolf GmbH). The dogs were placed under general anaesthesia, in lateral recumbent position [23] and their joints accessed via a medial approach. All dogs received appropriate NSAID therapy for peri- and post-operative pain.

### Elbow joint score system

Joints were scored as normal when the medial coronoid process (MCP) was observed unaltered in the arthroscopic, CT and radiographic assessments. Arthroscopic assessment was scored as pathologic if MCD was found (fissure, fragment or chondromalacia) [24]. CT images were scored as pathologic if changes in shape, attenuation, fragmentation or fissure line of the medial coronoid process were present and also if a trochlear notch sclerosis and irregular radio-ulnar joint space were observed. Joints evaluated radiographically were scored as pathologic if the outline was altered, and changes in shape or radio-density, an MCP fragment, or increased trochlear notch sclerosis were observed.

Two experienced observers (IG and HVB), blinded to the identity of the patient and results of the arthroscopic findings, evaluated randomly and in consensus the radiographic and CT images of each patient as paired sets (left- and right elbows). Imaging findings then were compared with the arthroscopic assessments.

### Statistical analysis

Statistical analysis was conducted with R (R Core Team, 2013). Sensitivity and specificity were calculated for CT compared with arthroscopy as the gold standard. In the smaller group of 141 dogs, CT, radiographs and arthroscopic results were compared (with arthroscopy considered to be the gold standard). In the large group of 180 dogs (141 dogs with CT, arthroscopy and radiographs + 39 dogs with radiographs and CT), CT was considered to be the reference test and compared with radiographs.

The reproducibility between the techniques was determined with Cohen’s Kappa. Cutoffs were used, as initially reported [25].

## Results

The study included 90 dogs, 40 female (44 %) and 50 male (56 %), with a median age of 27 months (range 5–133 months) and a median body weight of 29 kg (range 7–59 kg). Sixteen breeds were included, mostly Labrador retrievers (19 %), Golden retrievers (11 %), Bernese Mountain dogs (9 %) and Rottweiler’s (9 %) (Table 1). Bilateral elbow lameness was reported in 76 dogs (84 %) and unilateral lameness in 14 dogs (16 %).

**Table 1:** List of dog breeds included in the study

Breed	# of dogs
Labrador retriever	17
Golden retriever	10
Bernese mountain dog, Rottweiler	8
Mongrel	7
American staffordshire	4
Dogue de Bourdeaux, English Bulldog	3
Border Collie, Bouvier, Boxer, German shepherd, Münsterländer, Wetterhound	2
Argentino mastiff, Australian shepherd, Bassett, Beagle, Burbul, Cavalier king Charles, Chow Chow, Cocker spaniel, Staffordshire bull terrier, Fox terrier, Greater swiss mountain dog, Kromfhorländer, Landseer, Pug, Pyrenean shepherd, Schapendoes, St. Bernand, Viszla	1

A total of 180 elbow joints were evaluated with radiography and CT, 28 (16 %) were rated normal and 152 (84 %) as positive for MCD in the radiographic assessment; CT assessment showed 39 (22 %) non affected joints and 141 (78 %) positive for MCD (Table 2).

**Table 2:** Results for radiographic, CT and arthroscopic assessment for *n* = 141 and radiographic and CT assessment of *n* = 180

	Radiography (*n* = 141)	CT (*n* = 141)	Arthroscopy (*n* = 141)	Radiography (*n* = 180)	CT (*n* = 180)
Normal	11	13	14	28	39
MCD	130	128	127	152	141

**Fig. 1:**
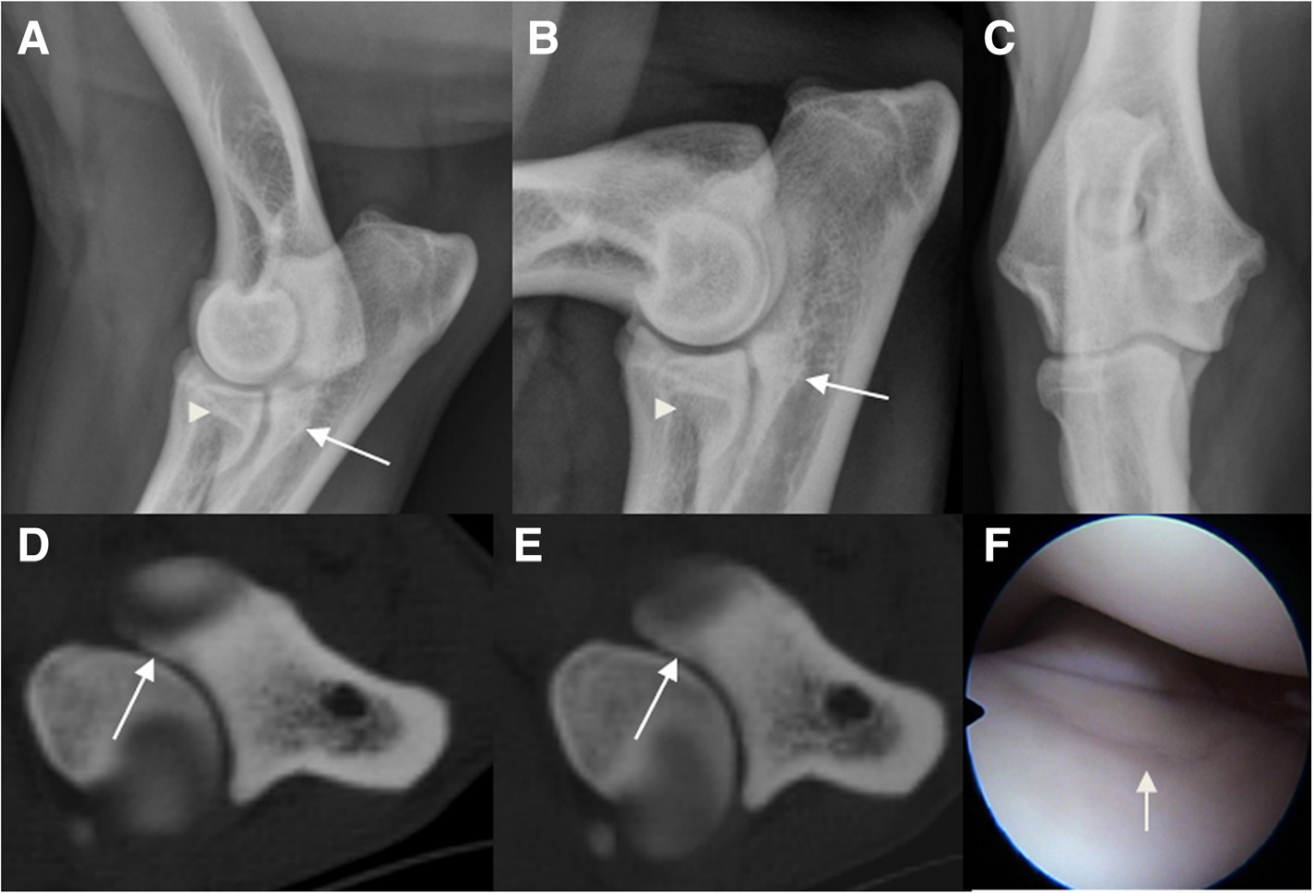
Radiographic, computed tomographic and arthroscopic findings of the canine elbow joint of a 20 month old Labrador retriever. Radiographic (latero-medial extended (**a**), latero-medial flexed (**b**), and cranio caudal (**c**) projections), computed tomographic (**d**-**e**) and arthroscopic (**f**) images of the left elbow joint from a male, 20 month old Labrador retriever. Radiographic images show subtrochlear sclerosis (*white arrow*) and the medial coronoid process is unclearly delineated with a heterogeneous density (*arrowhead*). The transverse CT images in bone algorithm at the level the medial coronoid process (**d**-**e**) demonstrate a hiperattenuated medial coronoid process with a fissure line and a non-displaced fragment (*white arrow*). On the arthroscopic image (**f**) of the same elbow joint, a fissure line of the articular cartilage of the medial coronoid process is visible (*white arrow*)

**Fig. 2:**
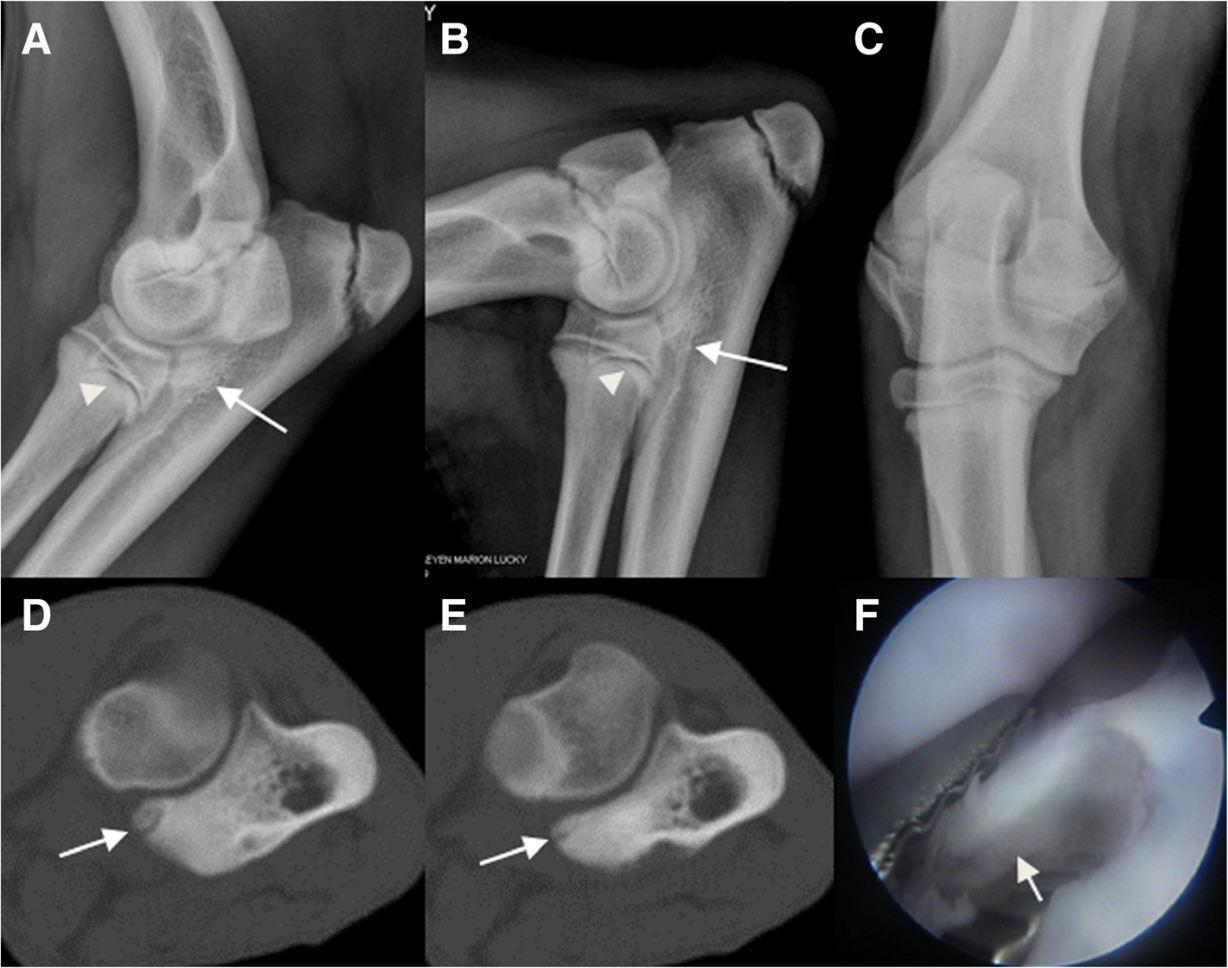
Radiographic, computed tomographic and arthroscopic findings of the canine elbow joint of a 5.5 month old Bernese mountain dog. Radiographic (latero-medial extended (**a**), latero-medial flexed (**b**), and cranio caudal (**c**) projections), computed tomographic (**d**-**e**) and arthroscopic (**f**) images of the left elbow joint from a male, 5.5 month old Bernese mountain dog. Radiographic images show subtrochlear sclerosis (*white arrow*) and the medial coronoid process is unclearly delineated (*arrowhead*). The transverse CT images in bone algorithm at the level the medial coronoid process (**d**-**e**) demonstrate the heterogeneous aspect of the medial coronoid process with a slightly-displaced fragment (*white arrow*). On the arthroscopic image (**f**) of the same elbow joint, a fragment of the medial coronoid process is visible (*white arrow*)

From the previous 180 joints a total of 141 joints were evaluated also with arthroscopy; in this group radiographic examination showed that 11 (8 %) were normal joints and 130 (92 %) were positive for MCD; in the CT assessment, 13 (9 %) were normal and 128 (91 %) were positive for MCD; arthroscopically 14 (10 %) were assessed as normal and 127 (90 %) as positive for MCD (Table 2) (Figs. 1 and 2).

**Fig. 3:**
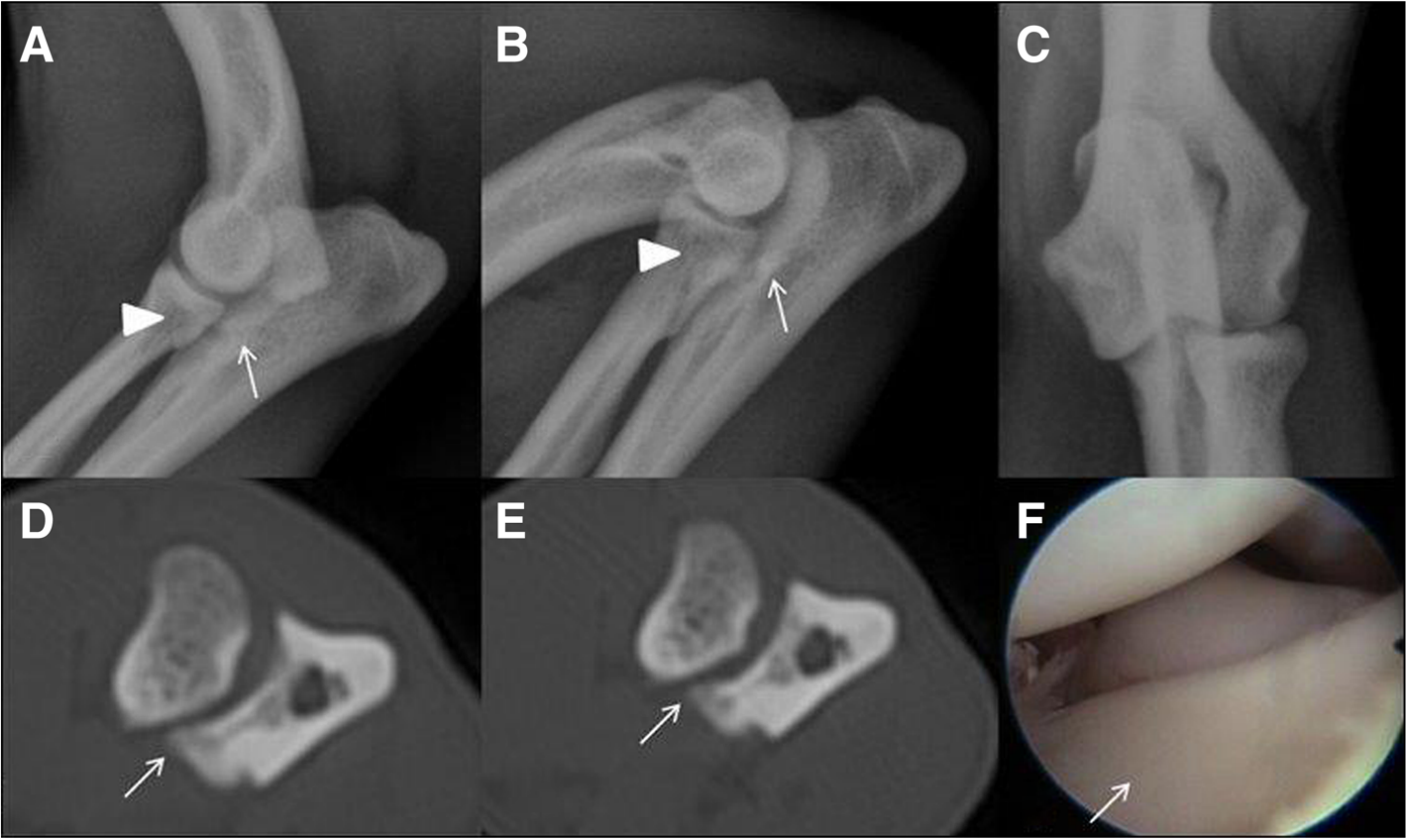
Radiographic, computed tomographic and arthroscopic findings of the canine elbow joint of a 12 month old Pug. Radiographic (latero-medial extended (**a**), latero-medial flexed (**b**), and cranio-caudal (**c**) projections), computed tomographic (**d**-**e**) and arthroscopic (**f**) images of the right elbow joint from a male, 12 month old Pug. Radiographic images show subtrochlear sclerosis (*white arrow*) and the medial coronoid process is unclearly delineated with a heterogeneous density (*arrowhead*). The transverse CT images in bone algorithm at the level the medial coronoid process (**d**-**e**) demonstrate the heterogeneous aspect of the medial coronoid process with a non-displaced fragment (*white arrow*). On the arthroscopic image (**f**) of the same elbow joint, a normal appearance of the medial coronoid process is visible (*white arrow*)

Sensitivity and specificity of CT using arthroscopy as the gold standard resulted in high values of 100 % of MCD for the former and 93 % for the latter (Table 2). One of 141 elbow joints was rated false positive on CT; none were rated false negative (Fig. 3).

The radiographic evaluation of the 141 elbow joints, compared with the CT findings, showed a sensitivity of 98 % and specificity of 69 %. A sensitivity of 98 % and specificity of 64 % were found in the total 180 joints (Table 3). With radiography, 14 of 180 elbow joints were scored false positive; three were scored false negative.

**Table 3:** Sensitivity and specificity values of CT and radiography

	CT // arthroscopy (*n* = 141)	Radiography // CT (*n* = 141)	Radiography // CT (*n* = 180)
Sensitivity	93 %	98 %	98 %
Specificity	100 %	69 %	64 %

Overall, the methods showed a high level of agreement (90–99 %), with an almost perfect agreement between CT and arthroscopy (kappa = 0.959) and less agreement between RX/CT, N = 141 (kappa = 0.72), and RX/CT, N = 180 (kappa = 0.69).

## Discussion

The distribution of our population was similar to previous studies [19, 26] with the lame dogs, more than half of whom were male, and with a majority of Labrador retriever, Golden retriever and Rottweiler breeds. However, our study included a larger variety of breeds, more animals and consequently a higher number of elbow joints (*n* = 180). The primary objective was to determine the sensitivity and specificity of CT, using arthroscopy as the gold standard. 141 of the 180 elbows were arthroscopically explored and the results were compared with the CT findings. Arthroscopy is considered the gold standard for assessment of cartilage lesions and MCD [27]; however, our results showed an almost perfect agreement between CT and arthroscopy.

All joints that presented a displaced fragment during arthroscopic assessment were correctly identified by CT. On the other hand, half of the non-displaced fragments diagnosed by CT were diagnosed as fissures during arthroscopy; this was due to the cartilage integrity found during the arthroscopic assessment of the joints, once this cartilage was probed a small number of joints (5) released a fragment of the MCP. One dog, a pug, was false positive, CT showed a non-displaced fragment which however arthroscopy found no evidence of such a lesion. This may have been related to the breed morphology or to the CT slice thickness of 1.3 mm. A smaller slice would provide a more representative image of the MCP in small breeds.

Several issues are important to produce reliable CT images. First, the image quality depends on the use of a protocol that avoids artefacts. This can be achieved using left lateral positioning [14], which avoids the presence of head and neck tissue in the gantry, because these tissues can cause streak artefacts that can potentially mimic fissure lines. The CT images should be made in a bone window setting and read using the correct window level that should be adapted when different slices are evaluated [28]. Experienced observers should read the images [26]. In this study, images of both elbows of each dog were evaluated together to ensure the joints were assessed accurately.

CT has been suggested as the more accurate technique for detecting primary MCD lesions compared with plain film radiography, xeroradiography, linear tomography and arthrography [9, 19]. However, a previous study that compared CT with arthroscopy found that CT can be contradictory [19]. Our findings with CT and during arthroscopic assessment of the joint expressed a perfect sensitivity of 100 %, which is higher than the values reported in another published study [19] where CT signs of dysplasia were associated with arthroscopic findings and a sensitivity of 71 % was presented. According to the latter study, the two techniques produced contradictory information about the presence of fragmentation of the medial coronoid process, which was attributed to the cartilaginous, rather than osteochondral, nature of some fragments.

CT specificity results in previous published studies presented a specificity of 84 % [19], 60.9 % [26] and 85 % [29]. However, in the latter study, the gold standard was arthrotomy, not arthroscopy. In our study, specificity resulted in a value of 93 %, because CT showed pathologic signs in one joint but no lesions were seen during arthroscopy. This may be due to our less aggressive arthroscopic approach; some non-displaced fragments may only become visible after aggressive probing of the cartilage surface of joints where MCD is suspected [19].

Missing purely cartilaginous MCP fragments [19] during CT assessment is also possible although less likely as most cartilage lesions will have a repercussion on the subchondral bone [30]. Nevertheless, CT has been recommended as a suitable imaging technique for early detection of MCD in Labrador retrievers from 14 weeks of age [31], because fragments originate at the MCP’s trabecular bone [32], most likely at its base [31]. One group has reported that the subchondral bone and the hyaline cartilage, physiologically and pathophysiologically, are one unit, and changes in the subchondral bone contribute further to the development of joint disease via pathologic changes in its biomechanical function [30]. The results of the present study demonstrate that an MCD lesion is not likely to be missed using high quality CT imaging technique, although lack of observer expertise [31] or inadequate imaging acquisition parameters and window level settings [28] could result in inadequately depicted lesions. Ideally histopathology should be used as gold standard to determine the accuracy of any evaluation of the elbow joint [33]; however this requires the evaluation of tissue samples, which was not suitable for our retrospective paper.

The high specificity, sensitivity and Cohen’s Kappa values of the CT assessment substantiated the decision to use the CT findings to determine the radiographic assessment sensitivity and specificity. This differs from a previous study that used the arthroscopic assessment [26] as its reference, but our study demonstrated the findings of the two groups with 141 and 180 elbow joints, respectively.

A 98 % radiographic sensitivity for both groups was obtained, which was similar to the 96.7 % obtained in a previous study [26] but higher than the 23.5 % obtained by Carpenter et al. in 1993. Experienced observers are said to increase the sensitivity [26]. Our reviewers’ broad experience and the appropriate management of CT image assessment led to the high sensitivity percentage values obtained using both radiographic and CT techniques. Having only two observers who made their diagnostics in consensus may have limited this study, however previous studies have used this modality to assess their findings [19, 34–37].

In human medicine [22] as well as in veterinary medicine [8, 19], arthroscopy is considered the gold standard for evaluating joint cartilage lesions. With arthroscopy only the articular surface can be evaluated and non-displaced fragments covered by intact cartilage could be missed. Our study demonstrates that the correlation between CT- and arthroscopic findings are almost perfect. Although it is stated that on CT cartilaginous lesions cannot be seen, most of the time pathological cartilage will have a repercussion on the underlying subchondral bone as the two structures can be considered as a union [30].

Nevertheless arthroscopy gives the opportunity of an immediate therapeutic intervention and assessment of soft tissue structures which imaging techniques such as radiography and CT do not offer. Moreover, recent imaging studies suggest the possibility of assessment of MCD signs in subchondral bone and articular cartilage by means of MRI [38, 39].

Specificity for radiography was 69 and 64 %, higher than the 40 % obtained in another study [26]. Nevertheless, a high percentage of false positives of 31 and 36 % for each group indicate that the radiographs were misread, possibly because there were many atypical breeds involved in the study; the 1.3 mm slice thickness in CT assessment may have led to loss of detail in smaller breeds; and finally because radiographic signs of degenerative joint disease do not always implicate fragments. In our study MCP delineation and density were the radiographic signs present in the false positive cases.

For this study, all the dogs had been referred due to forelimb lameness, so it can be assumed that the pathologic signs of MCD were more pronounced; moreover, not all joints with signs of MCD had arthroscopic assessment, since owners preferred to start a conservative therapy, rather than proceed with the arthroscopic intervention. Our results should not be extrapolated for screening purposes; the prevalence of MCD lesions would be lower in a screening population and is probably less severe as well.

## Conclusion

Our results show that CT, rather than arthroscopy, could be the main technique used to assess MCD lesions of the canine elbow joint, based on the high sensitivity and specificity obtained and the fact that arthroscopy cannot identify every fragment, especially the non-displaced fragments of the medial coronoid process. These results suggest that, when in doubt due to unclear radiographic signs of the MCP outline and density; or the trochlear notch sclerosis, CT could be used as a non-invasive technique to assess the presence of MCD.

## Abbreviations

CT: Computed tomography
FCP: Fragmented coronoid process
MCD: Medial coronoid disease
MCP: Medial coronoid process
MRI: Magnetic resonance imaging
